# Role of Kupffer cells in liver injury induced by CpG oligodeoxynucleotide and flucloxacillin in mice

**DOI:** 10.17179/excli2020-1103

**Published:** 2020-03-13

**Authors:** Yuying Gao, Binbin Song, Shigeki Aoki, Kousei Ito

**Affiliations:** 1 Laboratory of Biopharmaceutics, Graduate School of Pharmaceutical Sciences, Chiba University, Japan; 2 Key Laboratory of Ethnomedicine (Minzu University of China), Ministry of Education, School of Pharmacy, Minzu University of China, Beijing, China

**Keywords:** Kupffer cells, Toll-like receptor 9, Fas/Fas ligand, pro-inflammatory cytokines, mitochondrial permeability transition

## Abstract

CpG oligodeoxynucleotide (CpG-ODN) is a Toll-like receptor 9 (TLR9) agonist that can induce innate immune responses. In a previous study, flucloxacillin (FLUX; 100 mg/kg, gavage)-induced liver injury in mice was enhanced by co-administration of CpG-ODN (40 μg/mouse, intraperitoneally). In this study, the mechanism of CpG-ODN sensitization to FLUX-induced liver injury was further investigated in mice inhibited of Kupffer cells (KCs) function by gadolinium chloride (GdCl_3_; 10 mg/kg, intravenously). GdCl_3_-treated mice administrated with CpG-ODN and FLUX showed lower liver injury than wild-type (WT) mice treated with CpG-ODN and FLUX. Upregulation of Fas and FasL by CpG-ODN was also inhibited in GdCl_3_-treated mice and mitochondrial swelling in response to FLUX failed to occur regardless of pre-treatment with CpG-ODN. When FasL-mutant *gld/gld* mice were treated with CpG-ODN, mitochondrial swelling in response to FLUX was also inhibited. These results suggest that KCs play an essential role in liver injury induced by CpG-ODN and FLUX. CpG-ODN may activate KCs, resulting in induction of Fas/FasL-mediated apoptosis of hepatocytes. The Fas/FasL pathway may also be an upstream regulator of CpG-ODN- and FLUX-induced changes in mitochondrial permeability transition. These results enhance our understanding of the mechanism of the adjuvant effect of CpG-ODN in this mouse model of liver injury.

## Abbreviations

DILI: Drug-induced liver injury

KCs: Kupffer cells

TNF-α: Tumor necrosis factor-α

IL-6: Interleukin-6

IL-1β: Interleukin-1β

IFN-γ: Interferon-γ

IL-12: Interleukin-12

LPS: Lipopolysaccharide

MPT: Mitochondrial permeability transition

MPTPs: Mitochondrial permeability transition pores

ROS: Reactive oxygen species

FLUX: Flucloxacillin

ALT: Alanine aminotransferase

AST: Aspartate transaminase

T-Bil: Total bilirubin

CpG-ODN: CpG oligodeoxynucleotide

FasL: Fas ligand

NKT: Natural killer T

TLR9: Toll-like receptor 9

i.p.: Intraperitoneal injection

i.v.: Intravenous injection

H&E: Hematoxylin and eosin

BSA: Bovine serum albumin

## Introduction

Drug-induced liver injury (DILI) has become a significant hindrance to drug development (Watkins, 2011[42]). It is classified as intrinsic or idiosyncratic according to the clinical symptoms. In general, intrinsic DILI is dose-dependent and predictable, whereas idiosyncratic DILI is dose-independent, associated with immune responses, and challenging to detect in preclinical experiments and even during the clinical phase of drug development. Many experiments have been conducted *in vitro *and* in vivo* to clarify the mechanism of DILI. A potential mechanism may point to dysfunction of the immune system covering the up-regulation of innate immune responses induced by excessive inflammation (Jaeschke, 2006[18]), and adaptive immune responses (Gunawan and Kaplowitz, 2004[12]).

The recruitment of innate immune cells plays a crucial role in the initiation of adaptive immune responses. During infections in the liver, innate immune cells such as resident macrophages and dendritic cells firstly detect the presence of pathogens (bacteria, virus, damaged cells), and then release cytokines and chemokines to evoke a subsequent immune response (Liaskou et al., 2012[25]). Kupffer cells (KCs) are specialized macrophages in the liver, which account for approximately 20 % of the non-parenchymal cells (Duarte et al., 2015[9]; Racanelli and Rehermann, 2006[31]), as well as 80-90 % of the body's macrophages (Gregory and Wing, 1998[10]). After recognized with damage-associated molecular patterns or pathogens-associated molecular patterns, KCs are activated, and the pro-inflammatory cytokines, tumor necrosis factor-α (TNFα), interleukin-6 (IL-6), interleukin-1β (IL-1β), interferon-γ (IFN-γ), interleukin-12 (IL-12) are secreted, which will take part in the activation of immune response (Bilzer et al., 2006[5]; Schumann et al., 2000[34]). KCs are also confirmed the crucial role in some liver injury in mice. In the lipopolysaccharide (LPS)-induced liver injury which comes from Propionibacterium acnes, KCs are necessary for the LPS sensitization and the primary cells secreting interleukin-18, which induces the liver injury. Also, KCs contribute to concanavalin A-induced acute liver injury by inducing tissue factors, leading to the development of liver injury with endothelial damage (Tsutsui and Nishiguchi, 2014[39]). In a previous report, the mechanism of gut-derived bacterial products contributing to liver injury during chronic hepatitis B virus infection was clarified. In mice, the liver injury was dependent on interactions between NKT cells and KCs (Hou et al., 2017[16]). These studies suggest that KCs always take part in DILI.

The mechanism, mitochondrial dysfunction, is also concerned with bringing out the liver injury (Labbe et al., 2008[23]). Mitochondrial permeability transition (MPT) is a significant mechanism of drug-induced mitochondrial dysfunction (Jaeschke et al., 2012[19]). Generally, MPT is initiated by the opening of mitochondrial permeability transition pores (MPTPs) in the mitochondrial inner membrane (Baines and Gutierrez-Aguilar, 2018[2]; Biasutto et al., 2016[4]). Various factors enhance MPTPs opening, including oxidative stress and the presence of high levels of Ca^2+^ in the mitochondria (Mukherjee et al., 2019[28]). Although it is widely accepted that MPT can lead to mitochondrial swelling and cell death through apoptosis or necrosis (D'Arcy, 2019[6]; Karch et al., 2013[20]), the underlying mechanism of induction of MPT remains unclear. Drugs are thought to promote MPT by both direct and indirect mechanisms (Begriche et al., 2011[3]). Direct pathways include interference with MPTPs components and activation of endogenous MPTPs inducers. Indirect mechanisms include drug-induced oxidative stress, which results in the reactive oxygen species (ROS)-induced activation of MPTPs inducers (Jaeschke et al., 2012[19]).

Flucloxacillin (FLUX) is used for the treatment of Gram-positive infections. The incidence of FLUX-induced liver injury is estimated at 8.5 in every 100,000 new users in the United Kingdom (Russmann et al., 2005[33]). Several attempts to reproduce FLUX-induced liver injury in animals have been reported. CD4 T cell-depleted C57BL/6J mice treated with retinoic acid and FLUX show a mild and transient increase in serum alanine aminotransferase (ALT) and a dilated gallbladder, with no significant histological changes in the liver (Nattrass et al., 2015[29]). In our previous study, the CpG oligodeoxynucleotide (CpG-ODN)1826 was co-administrated with FLUX inducing hepatocyte apoptosis in mice. CpG-ODN is a Toll-like receptor 9 (TLR9) agonist, resulting in the initiation of innate immune responses that support the development of adaptive immunity (Hemmi et al., 2001[14]; Takeshita et al., 2001[38]). Based on this characteristic, CpG-ODN has been used as a vaccine adjuvant in many clinical trials as well as in experimental models (Al-Mariri et al., 2001[1]; Klinman, 2006[22]; Verthelyi et al., 2002[40]). In this mice model, CpG-ODN sensitized mice to FLUX-induced liver injury by activating natural killer T (NKT) cells to mediate Fas/Fas ligand (FasL)-dependent apoptosis against hepatocytes, while also sensitizing the hepatocytes to FLUX-induced mitochondrial dysfunction (Song et al., 2019[36]). However, the detailed underlying mechanism has not been identified.

Hence, in the present study, we investigated the involvement of KCs in the liver injury in mice treated with CpG-ODN and FLUX, and report the detailed mechanism through which CpG-ODN enhances susceptibility to MPT. These findings may promote our understanding of the KCs-involvement and MPT induction in DILI.

## Materials and Methods

### Animals

Female, 8-12-week-old C57BL/6J mice (Charles River Laboratories, Kanagawa, Japan), as well as FasL-mutant *gld/gld* mice in the C57BL/6J background and their counterpart C57BL/6J mice (Japan SLC, Shizuoka, Japan), were housed in an environment maintained at 25 °C with 3-70 % relative humidity and a 12 h light/dark cycle. Animals were treated humanely under the guidelines issued by the National Institutes of Health. All procedures were approved by the Animal Care Committee of Chiba University.

### Drug treatment

For co-treatment of mice with FLUX (Toronto Research Chemicals Inc., Toronto, Canada) and CpG-ODN 1826 (TCCATGACGTTCCTGACGTT) (Fasmac Co., Kanagawa, Japan), mice were fed FLUX (100 mg/kg) by oral gavage 5 h after intraperitoneal (i.p.) administration of CpG-ODN (40 μg/mouse). For inhibiting KCs' function, mice were pre-treated with gadolinium chloride (GdCl_3_; Wako, Osaka, Japan) [10 mg/kg, intravenously (i.v.)] 72 h before CpG-ODN administration (Kinoshita et al., 2010[21]; Lee et al., 2004[24]).

### Measurement of plasma ALT

ALT, a biomarker of liver injury, was measured 24 h after FLUX administration as described previously (Song et al., 2018[37]).

### Histology, immunohistochemistry, andterminal deoxynucleotidyl transferase dUTP nick end labeling (TUNEL) assay

After mice were sacrificed, liver sections were stained with hematoxylin and eosin (H&E) as previously described (Song et al., 2018[37]). A TUNEL staining kit (DeadEnd Fluorometric TUNEL System, Promega, Madison, WI, USA) was used to visualize apoptotic cells in liver sections.

### RNA isolation and quantitative real-time PCR

Total RNA was isolated and quantified as reported previously (Hirao et al., 2018[15]). GAPDH was used as the internal standard.

The following primers were used: TNF-α, forward 5′-TGGGACAGTGACCTGGACTGT-3′ and reverse 5′-TTCGGAAAGCCCATTTGAGT-3′; IL-6, forward 5′-TCGGAGGCTTAATTACACATGTTC-3′ and reverse 5′-TGCCATTGCACAACTCTTTTCT-3′; Fas, forward 5′-TATCAAGGAGGCCCATTTTGC-3′ and reverse 5′-TGTTTCCACTTCTAAACCATGCT-3′; FasL, forward 5′-TCCGTGAGTTCACCAACCAAA-3′ and reverse 5′-GGGGGTTCCCTGTTAAATGGG-3′; and GAPDH, forward 5′-ATGTGTCCGTCGTGGATCTG-3′ and reverse 5′-TGAAGTCGCAGGAGACAACC-3′.

### Isolation of liver mitochondria and measurement of MPT

Liver mitochondria were isolated and mitochondrial swelling (as an indicator of MPT) was determined according to a previously described method (Segawa et al., 2018[35]). Briefly, livers were isolated and placed in ice-cold isolation medium (250 mM sucrose, 10 mM HEPES-KOH (pH 7.2)) containing 0.5 mM EGTA, after which they were cut into small cubes with scissors, placed into 10 mL of medium, and homogenized 5 times in a Potter homogenizer. Homogenates were centrifuged at 770 × g for 5 min at 4 °C. The resulting supernatant was decanted and further centrifuged at 9,800 × g for 10 min at 4 °C. The supernatant was discarded, and the pellets were suspended in 10 mL of ice-cold isolation medium containing 0.3 mM EGTA. This suspension was centrifuged at 4,500 × g for 10 min at 4 °C, the supernatant was discarded, and the pellets were resuspended in 5 mL of ice-cold isolation medium containing 0.3 mM EGTA. Then, the suspension was centrifuged using a centrifugal force ranging from 2,000 × g for 2 min to 4,500 × g for 8 min and maintained at 4 °C. The final mitochondrial pellet was suspended in 150 μL of the ice-cold isolation medium. The protein concentration of the mitochondrial fraction was determined by the method of Lowry et al. (1951[26]) using a Multiskan JX system (MTX Lab Systems, Vienna, VA, USA).

Mitochondria (0.5 mg protein/mL) were preincubated in reaction medium (125 mM sucrose, 65 mM KCl, 5 mM succinate, and 10 mM HEPES-KOH (pH 7.4)) at 30 °C in the presence of 12.5 or 25 μM CaCl_2_. The reaction was initiated by the addition of various concentrations of FLUX (final 200-1,000 μM). Mitochondrial swelling as an indicator of MPT was determined by the decrease in absorbance at 540 nm at 30 °C on a UV-2550 spectrophotometer (Shimadzu, Kyoto, Japan).

### Statistical analysis

Data are presented as the mean ± standard error of the mean (SEM). Statistical comparisons were performed by two-way analysis of variants (ANOVA) (GraphPad Software, San Diego, USA). P-values < 0.05 were considered statistically significant.

## Results

### Involvement of KCs in hepatotoxicity induced by CpG-ODN and FLUX

**Figure 1:**
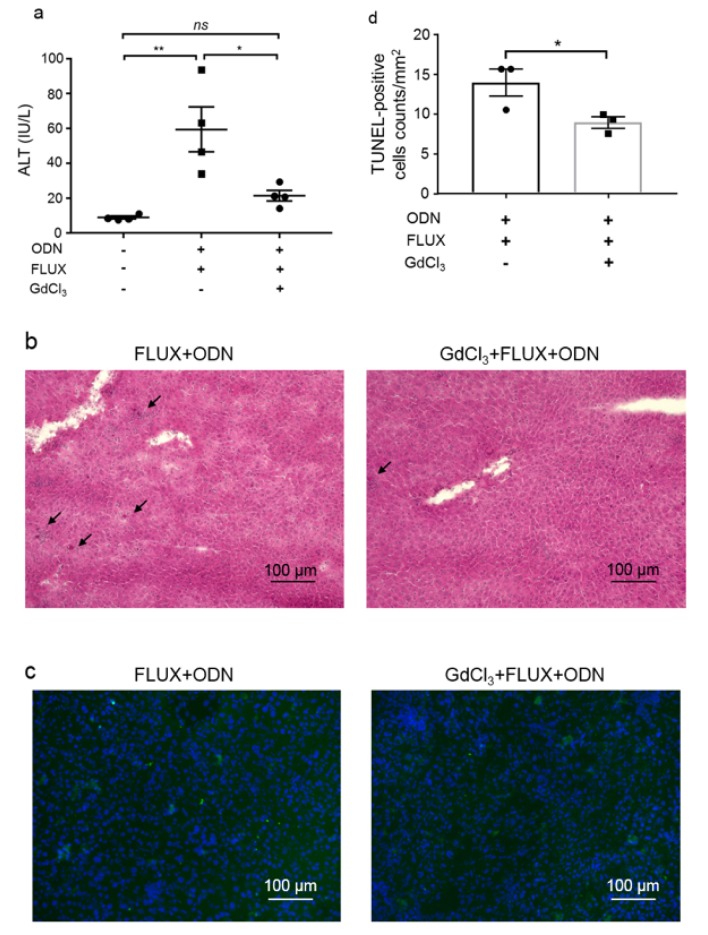
Liver injury induced by co-treatment of CpG-ODN and FLUX is suppressed by KCs inhibition. Mice (WT or GdCl_3_-treated) (Charles River Laboratories) were treated with FLUX (100 mg/kg, gavage) and CpG-ODN (40 μg/mouse, i.p.). a. Plasma ALT levels were measured 24 h after FLUX administration (n = 4). Data represent the mean ± SEM. *ns*, not significant. *p < 0.05 and **p < 0.01; two-way ANOVA. Liver sections from the mice were subjected to H&E staining (b) and TUNEL staining (c). In H&E staining images, arrows indicate apoptotic cells. In TUNEL staining images, green and blue represent TUNEL-positive cells and cell nuclei, respectively. Both images show representative results from three mice per group. Numbers of TUNEL-positive cells (d) were counted in 9 fields and calculated by per mm^2^ (n = 3). Data represent the mean ± SEM. *p < 0.05.

To investigate the involvement of KCs in CpG-ODN and FLUX-induced liver injury, mice were first injected with GdCl_3_ (10 mg/kg, i.v.) to inhibit KCs function. Three days after injection, mice [wild-type (WT) or GdCl_3_-treated] were treated with CpG-ODN (40 μg/mouse, i.p.) followed by administration of FLUX (100 mg/kg, gavage) 5 h later. As an indicator of liver damage, the plasma ALT level was evaluated 24 h after FLUX administration, as reported previously (Song et al., 2019[36]). Consistent with the previous study, increased ALT levels were found in mice co-treated with CpG-ODN and FLUX. By contrast, inhibition of KCs prevented the increase in plasma ALT levels observed in control mice co-treated with CpG-ODN and FLUX (Figure 1a(Fig. 1)).

Furthermore, liver sections were subjected to histological and TUNEL staining to characterize the liver injury. With H&E staining, apoptotic cells (arrows) were observed in the livers of WT mice co-treated with CpG-ODN and FLUX, whereas in GdCl_3_-treated mice, the number of apoptotic cells in the liver was greatly reduced (Figure 1b(Fig. 1)). TUNEL staining to label apoptotic cells was consistent with the histological findings (Figure 1c and d(Fig. 1)), and these observations were consistent with the liver injury evidenced by the increased plasma ALT levels. These results demonstrate that KCs are indispensable for CpG-ODN sensitization to FLUX-induced liver injury in mice.

### KCs-inhibition, CpG-ODN-induced immune responses

**Figure 2:**
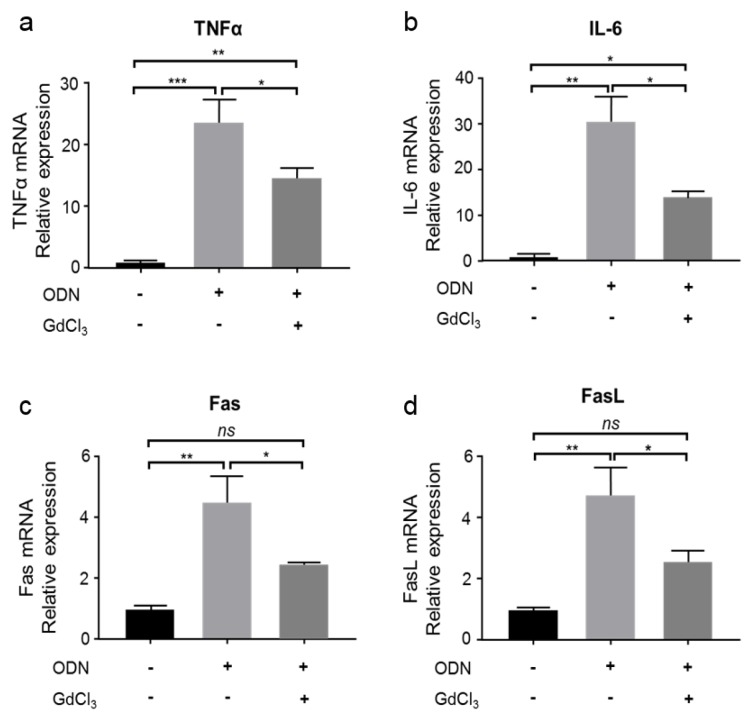
KCs produce pro-inflammatory cytokines and activate Fas/FasL-mediated apoptosis. CpG-ODN (40 μg/mouse, i.p.) was administered to WT or GdCl_3_-treated mice (Charles River Laboratories). Five hours later, the liver homogenate was analyzed for the relative mRNA expression of TNF-α (a), IL-6 (b), Fas (c), and FasL (d). n = 3. Data represent the mean ± SEM. *ns*, not significant. *p < 0.05, **p < 0.01, and ***p < 0.001; two-way ANOVA

It has been reported that KCs respond to CpG-ODN and promote NKT cell activation in hepatitis B surface antigen transgenic mice (Hou et al., 2017[16]). To further verify the involvement of KCs in CpG-ODN-mediated immune responses, which sensitize mice to FLUX-induced liver injury, mice (WT and GdCl_3_-treated) were administrated with CpG-ODN (40 μg/mouse, i.p.). The relative mRNA expression of pro-inflammatory cytokines, TNF-α and IL-6, was measured 5 h after CpG-ODN administration. As shown in Figure 2a and 2b(Fig. 2), the mRNA expression of both TNF-α and IL-6 was increased by CpG-ODN treatment. When mice were pre-treated with GdCl_3_, although a partial increase in the expression of TNF-α and IL-6 by CpG-ODN was observed, this increase was significantly less than that of mice without KCs inhibition (Figure 2a and 2b(Fig. 2)), simultaneously indicating KCs are prime source cells of TNF-α and IL-6.

The expression of Fas and FasL was also measured. Similar to the expression of TNF-α and IL-6, CpG-ODN treatment increased the mRNA expression of Fas and FasL compared with the control group (Figure 2c and 2d(Fig. 2)), but the induction of Fas and FasL was inhibited in GdCl_3_-treated mice administrated with CpG-ODN (Figure 2c and 2d(Fig. 2)). These results suggest that KCs inhibition suppresses the induction of Fas/FasL-mediated apoptosis in response to CpG-ODN.

Collectively, these results suggest that CpG-ODN acts on KCs to initiate an innate immune response characterized by increased expression of TNF-α and IL-6, and increased induction of Fas/FasL-mediated apoptosis.

### CpG-ODN sensitization to FLUX-induced mitochondrial swelling was suppressed in GdCl_3_-treated mice

We have shown that KCs play an essential role in CpG-ODN-induced innate immune responses (Figure 2a-2d(Fig. 2)). In our previous study, we found that CpG-ODN sensitized mice to FLUX-induced mitochondrial swelling in an MPT-dependent manner (Song et al., 2019[36]). Therefore, in this study, we hypothesized that KCs might be involved in the FLUX-induced MPT and investigated the effect of KCs inhibition on MPT induction.

**Figure 3:**
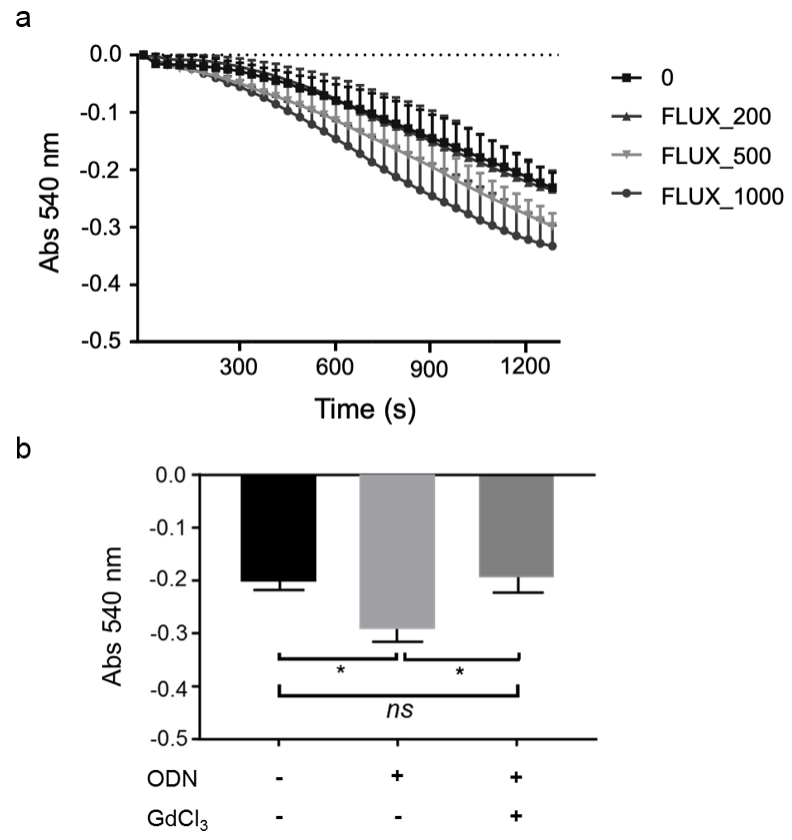
KCs are involved in CpG-ODN sensitization to FLUX-induced mitochondrial swelling. a. Mitochondrial swelling curve. Liver mitochondria from WT mice were exposed to 200, 500, and 1000 μM FLUX with 12.5 μM Ca^2+^. Mitochondrial swelling was determined by the decrease in absorbance at 540 nm. Data represent the mean ± SEM (n = 3). b. Liver mitochondria were isolated from WT and GdCl_3_-treated mice (Charles River Laboratories) 5 h after treatment of mice with CpG-ODN (40 μg/mouse, i.p.) or vehicle. Mitochondrial swelling was determined by the decrease in absorbance at 540 nm. The absorbance 1,200 s after exposure to 200 μM FLUX with 12.5 μM Ca^2+ ^is shown. Data represent the mean ± SEM (n = 3). *ns*, not significant. *p < 0.05; two-way ANOVA

Mice (WT or GdCl_3_-treated) were fasted overnight. Liver mitochondria were isolated 5 h after the administration of CpG-ODN or vehicle, and then exposed to 0, 200, 500, or 1,000 μM FLUX to investigate mitochondrial swelling. Consistent with our previous study, in WT mice, exposure to FLUX induced MPT in a concentration-dependent manner (Figure 3a(Fig. 3)). As shown in Figure 3b(Fig. 3), exposure to 200 μM FLUX could induce stronger MPT in the isolated mitochondria of WT mice treated with CpG-ODN than in those from the control group. By contrast, KCs inhibition reduced the mitochondrial swelling to almost the same level as in the control mice, regardless of CpG-ODN pre-treatment (Figure 3b(Fig. 3)). These results suggest that CpG-ODN sensitization to FLUX-induced MPT was also dependent on KCs.

### Fas/FasL-mediated apoptosis was required for CpG-ODN-sensitization to FLUX-induced MPT

Above, we show that KCs mediate the production of pro-inflammatory cytokines in response to CpG-ODN (Figure 2a and 2b(Fig. 2)) and are also involved in the upregulation of Fas and FasL in the liver (Figure 2c and 2d(Fig. 2)). Meanwhile, by KCs inhibition, CpG-ODN sensitization to FLUX-induced mitochondrial swelling was diminished (Figure 3b(Fig. 3)). However, the mechanism underlying this result was unclear. Here, we hypothesized that the MPT in this mouse model was mediated by KCs through the induction of Fas/FasL-mediated apoptosis. This hypothesis was tested using *gld/gld* (FasL-mutated) and WT mice.

**Figure 4:**
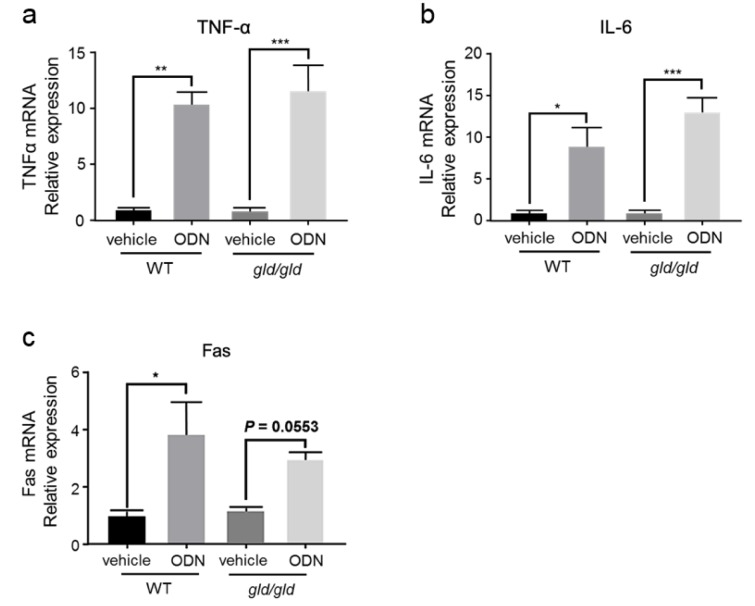
The expression of pro-inflammatory cytokines and Fas is increased by CpG-ODN in both WT and *gld/gld* mice. WT and *gld/gld* mice (Japan SLC) were treated with or without CpG-ODN (40 μg/mouse, i.p.). Five hours later, the liver homogenate was analyzed for the relative mRNA expression of TNF-α (a), IL-6 (b), and Fas (c). n = 3. Data represent the mean ± SEM. *ns* not significant. *p < 0.05, **p < 0.01, and ***p < 0.001; two-way ANOVA

First, *gld/gld* and WT mice were treated with CpG-ODN, and the relative mRNA expression of TNF-α, IL-6, and Fas in the liver was measured 5 h later. As shown in Figure 4(Fig. 4), the expression of TNF-α (Figure 4a(Fig. 4)) and IL-6 (Figure 4b(Fig. 4)) increased in *gld/gld* mice after ad-ministration of CpG-ODN, consistent with the response to CpG-ODN in WT mice. Although FasL is mutated in *gld/gld* mice, the expression of Fas on hepatocytes was also slightly upregulated, although this was not statistically significant (Figure 4c(Fig. 4)).

**Figure 5:**
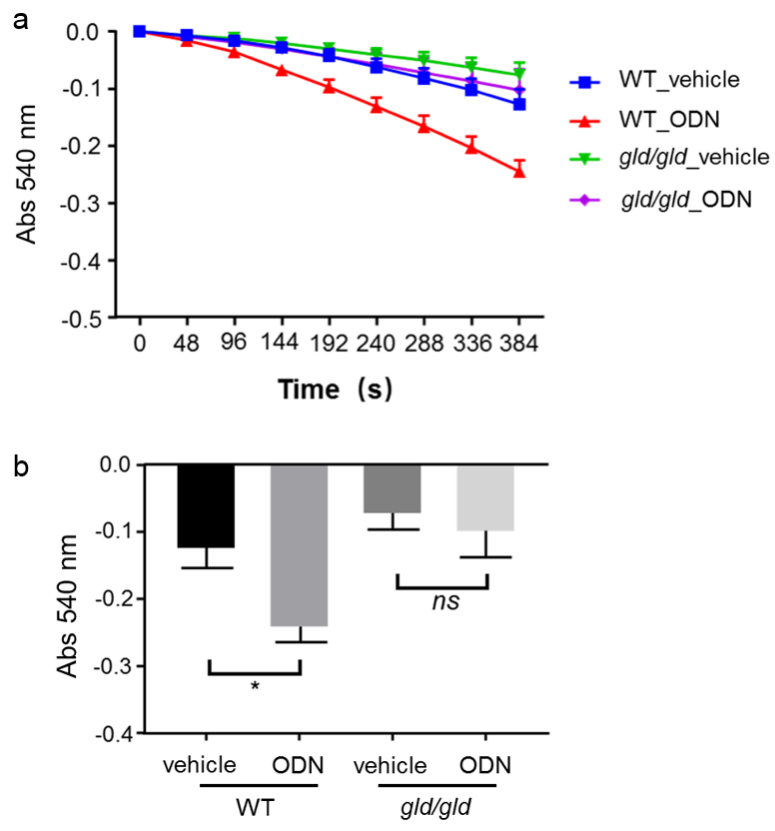
Fas/FasL-mediated apoptosis is critical for CpG-ODN sensitization to FLUX-induced mitochondrial swelling. Mitochondria from WT and *gld/gld* mice (Japan SLC, Inc.) were isolated 5 hours after CpG-ODN treatment (40 μg/mouse, i.p.), and then exposed to 200 μM FLUX with 25 μM Ca^2+^. Mitochondrial swelling was determined by the decrease in absorbance at 540 nm. a. Mitochondrial swelling curve. Data represent the mean ± SEM (n = 3). b. Mitochondrial swelling at 384 s. Data represent the mean ± SEM (n = 3).* ns*, not significant. *p < 0.05; two-way ANOVA

Next, liver mitochondria were exposed to 200 μM FLUX in the same time course as in the previous swelling assay. As a positive control, isolated mitochondria from WT mice pre-treated with CpG-ODN showed a stronger MPT response compared with those from vehicle control mice (Figure 5a and 5b(Fig. 5)). However, in *gld/gld* mice, mitochondrial swelling in the CpG-ODN pre-treatment group was equal to that in the vehicle control group (Figure 5a and 5b(Fig. 5)). These results confirm that the induction of MTP in CpG-ODN-treated mice is dependent on the Fas/FasL pathway.

## Discussion

The mechanism of FLUX-induced liver injury is complex and diverse. Apart from genetic factors, increasing age and females suggest an increased risk of injury (Russmann et al., 2005[33]; Wing et al., 2017[44]). In our previous study, CpG-ODN, a TLR9 agonist, was used to sensitize female mice to FLUX-induced liver injury (Song et al., 2019[36]). In this mouse model, the acute liver injury detected 24 h after FLUX administration was characterized by innate immune responses and MPT, which is significantly associated with mitochondrial dysfunction. However, the detailed mechanisms involved in this response had not been elucidated. Therefore, in this study, we further investigated the mechanisms of CpG-ODN sensitization to FLUX-induced liver injury.

Many cells have been reported to express TLR9, including endothelial cells, hepatic dendritic cells, KCs (Wu et al., 2010[45]), and hepatic stellate cells (Watanabe et al., 2007[41]). Among them, KCs play a significant role in clearing bacteria and foreign proteins from the circulation. It has been reported that KCs promote liver disease and DILI by activating the innate immune system. Inhibition of KCs suppresses hepatic steatosis, indicating that activation of KCs plays a crucial role in non-alcoholic fatty liver disease pathogenesis and progression (Huang et al., 2010[17]). Another report showed that liver injury during alcoholic liver disease was dependent on activation of KCs by endotoxin, which is released by bacteria living in the intestine at the time of alcohol consumption (Wheeler, 2003[43]). Notably, FLUX is used to treat bacterial infections. Synthetic CpG-ODN, which mimics microbial genomes, has also been used to mimic bacterial infection. Given the role of the gut-liver axis in KCs activation, in our study we investigated the involvement of KCs in liver injury. We found that inhibition of KCs effectively attenuated liver injury induced by co-treatment of CpG-ODN and FLUX (Figure 1(Fig. 1)).

Activated KCs release cytokines. These cytokines play an essential role in modulating immune responses. Among these, TNF-α and IL-6 (Gregory et al., 1998[11]; Helk et al., 2013[13]; Luster et al., 1994[27]) are largely reported contributing to the liver injury. For instance, TNF-α released by activated KCs contributed to liver destruction in mice with amebic liver abscess (Helk et al., 2013[13]). IL-6 was produced by KCs early during Listeria infection (Gregory et al., 1998[11]). Furthermore, in a mouse osteoblastic cell line, a combination of TNF-α, IL-1β, and IFN-γ increased the expression of Fas mRNA and protein, and led to Fas/FasL-mediated apoptosis (Ozeki et al., 2002[30]). In our study, we found that co-treatment of mice with CpG-ODN and FLUX increased the expression of TNF-α, IL-6, Fas, and FasL, and this response was significantly, although not completely, prevented by inhibition of KCs (Figure 2a-d(Fig. 2)). The incomplete suppression of this response is likely due to residual KCs or other TLR9-expressing cells in the liver responding to CpG-ODN (Yang and Seki, 2012[46]).

MPT plays a central role in apoptosis and necrosis (Desagher and Martinou, 2000[7]). Previously, we found that CpG-ODN sensitized liver mitochondria to MPT induced by FLUX (200 μM) (Song et al., 2019[36]). In this study, we found that the increased sensitivity of CpG-ODN-treated mice to MPT was abrogated by KCs inhibition (Figure 3b(Fig. 3)). Apoptosis can be initiated via two signaling pathways: the intrinsic pathway, which originates from mitochondria, and the extrinsic pathway, which originates from death signaling (Dinh et al., 2015[8]; Roy and Nicholson, 2000[32]). These two pathways cross-talk in the execution of cell death (Roy and Nicholson, 2000[32]). Induction of apoptotic signaling pathways leads to activation of caspase-8, which cleaves Bid or other Bcl2 family proteins that promote mitochondrial permeabilization. To clarify whether activation of the Fas/FasL-mediated apoptotic pathway was involved in the increased MPT in CpG-ODN-sensitized FLUX-induced liver injury, FasL-mutant *gld/gld* mice were used. There was a similar degree of induction of TNF-α and IL-6 in *gld/gld* mice compared with WT mice manifesting the activation of KCs step ahead the activation of Fas/FasL-mediated pathway (Figure 4a-b(Fig. 4)). However, in FasL-mutant mice pre-treated with CpG-ODN, mitochondrial swelling was equivalent to that seen in vehicle control mice exposed to FLUX (200 μM), while MPT increased in WT mice pre-treated with CpG-ODN. These findings suggest that the effect of CpG-ODN on FLUX-induced MPT was dependent on induction of Fas/FasL-mediated apoptosis.

Taken together, these results indicate that CpG-ODN enhanced FLUX-induced liver injury by activating KCs, resulting in production of pro-inflammatory cytokines (TNF-α and IL-6) and initiation of Fas/FasL-mediated apoptosis, but was insufficient to induce liver injury without FLUX administration. When FLUX was administered to mice pre-treated with CpG-ODN, Fas/FasL-mediated apoptosis contributed to FLUX-induced MPT, ultimately inducing liver injury. However, we do not yet know whether activation of the Fas/FasL pathway is dependent on the release of pro-inflammatory cytokines. In the future, we propose to investigate the association between cytokines release and Fas/FasL pathway induction by blockade of cytokines with semapimod, a pharmacological inhibitor of cytokine production, as well as to investigate the specific relationship between TNF-α or IL-6 and the Fas/FasL pathway using blocking antibodies.

In this study, we report the detailed mechanism through which CpG-ODN sensitizes mice to FLUX-induced liver injury. These findings may enhance our understanding of the complex pathogenesis of drug-induced acute liver injury in the clinic.

## Notes

Yuying Gao and Binbin Song contributed equally as first authors.

## Conflicts of interest

The authors have no conflicts of interest to report.

## Acknowledgements

This work was supported by a Japan Society for the Promotion of Science KAKENHI grant (number 19H03386).
